# Difference in Mono-O-Glucosylation of Ras Subtype GTPases Between Toxin A and Toxin B From *Clostridioides difficile* Strain 10463 and Lethal Toxin From *Clostridium sordellii* Strain 6018

**DOI:** 10.3389/fmicb.2018.03078

**Published:** 2018-12-21

**Authors:** Harald Genth, Johannes Junemann, Chantal M. Lämmerhirt, Arlen-Celina Lücke, Ilona Schelle, Ingo Just, Ralf Gerhard, Andreas Pich

**Affiliations:** Institute of Toxicology, Hannover Medical School, Hanover, Germany

**Keywords:** Rho, Ras, GTPase, large clostridial glucosylating toxins, MRM analysis

## Abstract

*Clostridioides difficile* toxin A (TcdA) and Toxin B (TcdB) trigger inflammasome activation with caspase-1 activation in cultured cells, which in turn induce the release of IL-6, IFN-γ, and IL-8. Release of these proinflammatory responses is positively regulated by Ras-GTPases, which leads to the hypothesis that Ras glucosylation by glucosylating toxins results in (at least) reduced proinflammatory responses. Against this background, data on toxin-catalyzed Ras glucosylation are required to estimate of pro-inflammatory effect of the glucosylating toxins. In this study, a quantitative evaluation of the GTPase substrate profiles glucosylated in human colonic (Caco-2) cells treated with either TcdA, TcdB, or the related *Clostridium sordellii* lethal toxin (TcsL) was performed using multiple reaction monitoring (MRM) mass spectrometry. (H/K/N)Ras are presented to be glucosylated by TcsL and TcdA but by neither TcdB isoform tested. Furthermore, the glucosylation of (H/K/N)Ras was detected in TcdA-(not TcdB)-treated cells, as analyzed exploiting immunoblot analysis using the Ras glucosylation-sensitive 27H5 antibody. Furthermore, [^14^C]glucosylation of substrate GTPase was found to be increased in a cell-free system complemented with Caco-2 lysates. Under these conditions, (H/K/N)Ras glucosylation by TcdA was detected. In contrast, TcdB-catalyzed (H/K/N)Ras glucosylation was detected by neither MRM analysis, immunoblot analysis nor [^14^C]glucosylation in a cell-free system. The observation that TcdA (not TcdB) glucosylates Ras subtype GTPases correlates with the fact that TcdB (not TcdA) is primarily responsible for inflammatory responses in CDI. Finally, TcsL more efficaciously glucosylated Ras subtype GTPase as compared with TcdA, reinforcing the paradigm that TcsL is the prototype of a Ras glucosylating toxin.

## Introduction

The family of large clostridial glucosylating toxins (LCGTs) encompasses toxin A (TcdA) and toxin B (TcdB) of *Clostridioides difficile*, lethal toxin (TcsL) and hemorrhagic toxin (TcsH) from *Clostridium sordellii*, and large toxin (TpeL) from *C. perfringens* (Popoff and Bouvet, 2009; Genth and Just, 2011; Genth et al., 2014; Jank et al., 2015). These toxins exhibit molecular masses ranging from 191 to 307 kDa and an AB-like domain structure with a N-terminal glucosyltransferase domain and a C-terminal delivery domain. Upon cell entry by receptor-mediated endocytosis, the LCGTs mono-O-glucosylate Rho/Ras subfamily GTPases (D’Urzo et al., 2012; Genth et al., 2016). Rho and Ras subtype proteins are key regulators of cytoskeletal dynamics, cell proliferation, and cell death/survival. Mono-O-glucosylation of RhoA at Thr-37 or of Rac/Cdc42 and (H/K/N)Ras at equivalent Thr-35 renders cellular Rho/Ras proteins inactive, resulting in a breakdown of the actin cytoskeleton, inhibition of cell proliferation, and cell death (Dreger et al., 2009; Lica et al., 2011; Farrow et al., 2013; May et al., 2013; Wohlan et al., 2014). The glucosylating toxins are regarded to be responsible for the loss of intestinal barrier function and for inflammation observed in *C. difficile*-associated diarrhea (CDAD), *C. sordellii*-induced haemorrhagic enteritis and enterotoxemia, and *C. perfringens*-associated necrotic enteritis (Popoff, 2011; Smits et al., 2016).

In cultured cells, TcdA and TcdB trigger inflammasome activation with caspase-1 activation, based on the recognition of RhoA glucosylation by pyrin (Ng et al., 2010; Xu et al., 2014). Caspase-1 subsequently activates IL-1β and IL-18, which in turn induce the release of IL-6, IFN-γ, and IL-8. Either of these proinflammatory responses is positively regulated by Ras-GTPases (Sparmann and Bar-Sagi, 2004; Johnson and Chen, 2012), which leads to the hypothesis that Ras glucosylation by glucosylating toxins results in (at least) reduced proinflammatory responses. Some observations support this hypothesis, as TcdB (not the Ras glucosylating TcdA) has been suggested to be primarily responsible for inflammatory responses in CDI (Carter et al., 2015; Popoff, 2017). Against this background, data on Ras glucosylation are required to estimate the proinflammatory effects of the toxins. A quantitative evaluation of the GTPase substrate profiles glucosylated in cells treated with TcdB (as well as with the related TcsL) is not yet available. Therefore, the profiles of substrate GTPases glucosylated by TcdB, TcsL, and TcdA were evaluated in toxin-treated Caco-2 cells using multiple reaction monitoring (MRM) mass spectrometry. MRM analysis allows the quantification of the glucosylation of small GTPases in cultured cells (Junemann et al., 2017). This study provides evidence on the glucosylation of (H/K/N)Ras and Rap(1/2) by TcsL and TcdA. In contrast, neither TcdB isoform tested glucosylated (H/K/N)Ras and Rap(1/2). Furthermore, the glucosylation of (H/K/N)Ras was detected in TcdA-treated cells, as analyzed exploiting the glucosylation-sensitive Ras(Mab 27H5) antibody (Huelsenbeck et al., 2009). Finally, [^14^C]glucosylation of (H/K/N)Ras by TcdA was found in a cell-free system complemented with Caco-2 lysates.

## Results

### TcdA-Catalyzed Glucosylation of Ras Subtype GTPases in Cultured Cells

**FIGURE 1:**
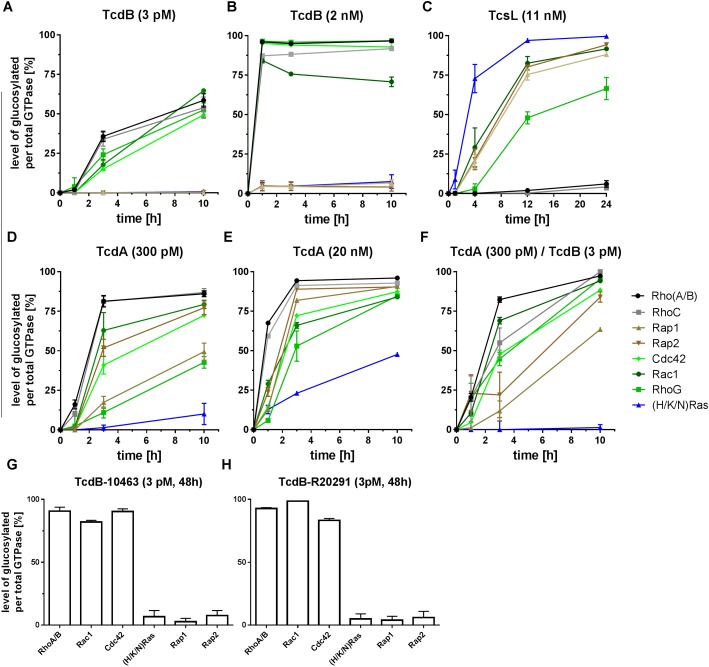
Mass spectrometry-based evaluation of the substrate GTPase profiles glucosylated by LCGTs. Caco-2 cells were exposed to recombinantly prepared TcdB-10463 **(A,B,F,G)**, to TcsL prepared from *C. sordellii* strain 6018 (TcsL-6018) **(C)**, recombinantly prepared TcdA-10463 **(D–F)**, and recombinantly prepared TcdB-20291 **(H)**. Upon cell lysis, small GTPases of the Rho and Ras subfamilies were analyzed for cellular concentrations of glucosylated GTPases using MRM analysis. Glucosylation was given as the ratio of the concentration of glucosylated GTPase per concentration of total GTPase. All experiments were conducted with three biological replicates. The error bars are representing the SD of the mean.

The *C. difficile* strain VPI10463 [isolated from an abdominal wound, (Theriot et al., 2011)] has long been regarded as a reference strain. *C. difficile* strain VPI10463 exhibits an A+B+CDT- toxinotype i.e., it produces TcdA-10463 and TcdB-10463 but not the binary *C. difficile* toxin (CDT) (Genth et al., 2008). To evaluate possible differences in the GTPase substrate profiles of TcdA-10463 and TcdB-10463, Caco-2 cells were treated with the toxins and GTPase substrate profiles were analyzed in terms of the MRM method. Treatment of Caco-2 cells with TcdB-10463 resulted in time-dependent mono-O-glucosylation of the Rho subtype GTPases Rho(A/B/C), Rac1, RhoG, and Cdc42 (Figure 1A). Remarkably, neither Rap(1/2) nor (H/K/N)Ras were glucosylated (Figure 1A). Furthermore, TcdB-10463-catalyzed glucosylation of Rap(1/2) or (H/K/N)Ras was observed neither upon treatment with an about three orders of magnitude higher TcdB-10463 concentration of 2 nM (Figure 1B) nor upon prolonged TcdB-10463 treatment for 48h (Figure 1G). TcdA-10463 glucosylated Rap(1/2) and (less efficaciously) (H/K/N)Ras as well as its canonical Rho subfamily substrate GTPases including Rho(A/B/C), Rac1, RhoG, and Cdc42 (Figures 1D,E). The latter observations were consistent with published data (Junemann et al., 2017). Combined treatment of Caco-2 cells with TcdA (300 pM) and TcdB (3 pM) resulted in glucosylation kinetics almost similar to that of TcdA (300 pM) alone, excluding synergistic effects in the kinetics of substrate GTPase glucosylation upon combined toxin treatment. The only exception was (H/K/N)Ras, which glucosylation seemed to suppressed upon combined treatment with TcdA and TcdB (Figures 1D,F). The latter observation suggests that TcdB suppressed TcdA-catalyzed Ras glucosylation.

Next, the substrate GTPase profile of TcdB from the hypervirulent, toxinotype A+B+CDT+ *C. difficile* strain R20291 (isolated from the feces of a symptomatic patient in United Kingdom) was evaluated upon prolonged treatment of Caco-2 cells for 48 h. TcdB-R20291 specifically glucosylated Rho/Rac/Cdc42 subtype GTPases (but not Ras subtype GTPases) (Figure 1H). TcdB-R20291 and TcdB-10463 thus exhibited a similar substrate GTPase profile, with the Ras subtype GTPases Rap(1/2) and (H/K/N)Ras not being glucosylated (Figures 1G,H).

Among the family of glucosylating toxins, *C. sordellii* lethal toxin (TcsL) has been classified as the prototype of Ras glucosylating toxin (Genth and Just, 2011; Genth et al., 2014). This notion was re-confirmed using MRM analysis of TcsL-treated Caco-2 cells: Ras subtype GTPases Rap(1/2) and (H/K/N)Ras were the preferred cellular substrates of the related TcsL: (H/K/N)Ras, Rac1, Rap(1/2) > RhoG > > Rho(A/B/C) (Figure 1C). Weak glucosylation of Rho(A/B/C) (not observed until TcsL treatment for 24 h) was background (Figure 1C). In contrast to TcdA/TcdB-treated Caco-2 cells, Cdc42 was not read from TcsL-treated Caco-2 cells, which might due to Cdc42 degradation (Figure 1C).

**FIGURE 2:**
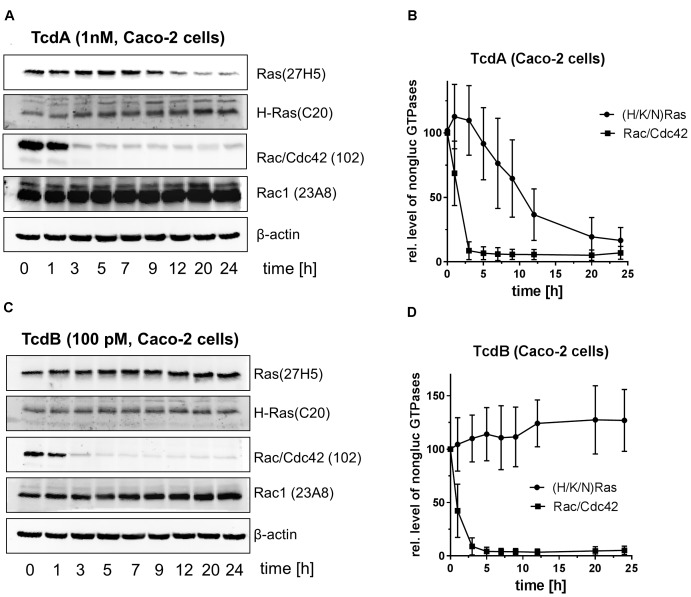
Immunoblot-based analysis of the glucosylation of (H/K/N)Ras and Rac/Cdc42 in TcdA- and TcdB-treated Caco-2 cells. Caco-2 cells were exposed to natively prepared TcdA-10463 **(A,B)** or TcdB-10463 **(C,D)** for the indicated times. Upon cell lysis, the cellular concentrations of glucosylated (H/K/N)Ras and Rac/Cdc42 subtype GTPases were evaluated using immunoblot analysis. Immunoblots were quantified using Kodak software. The amounts of non-glucosylated Rac/Cdc42 and non-glucosylated (H/K/N)Ras relative to the respective total levels of Rac1 and H-Ras are expressed as mean ± SD of three experiments.

Immunoblot analysis exploiting the Rac1(Mab 102) and the Ras(Mab 27H5) antibodies have evolved into a routine method for tracking mono-O-glucosylation of cellular Rac/Cdc42 and (H/K/N)Ras, respectively (Genth et al., 2006; Huelsenbeck et al., 2009; Brandes et al., 2012). Once Rac/Cdc42 or (H/K/N)Ras is mono-O-glucosylated, the antibodies do not detect their epitopes, resulting in signal loss. In contrast, the H-Ras (C20) and the Rac1 (Mab 23A8) antibodies are glucosylation insensitive and can be used to quantify total levels of H-Ras or Rac1, respectively. TcdA-10463-treated Caco-2 cells exhibited time-dependent glucosylation of (H/K/N)Ras with about 50% of total (H/K/N)Ras being glucosylated after 12 h (Figures 2A,B). Detection of exemplarily H-Ras using the H-Ras(C20) antibody showed no decrease, indicating that the cellular level of H-Ras was not changed upon TcdA treatment (Figure 2A). In contrast to (H/K/N)Ras, TcdA-10463-catalyzed glucosylation of Rac/Cdc42 was almost complete upon 3 h of TcdA treatment, as evaluated using the Rac1(clone 102) antibody (Figures 2A,B). The cellular level of Rac1 was not changed as analyzed using the Rac1(Mab 23A8) antibody, confirming that decreasing detection by the Rac1(Mab 102) antibody was due to glucosylation but not due to degradation (Figure 2A). In TcdB-10463-treated Caco-2 cells, rapid glucosylation of Rac/Cdc42 but not of (H/K/N)Ras was detected exploiting the Rac1(Mab 102) and the Ras(Mab 27H5) antibodies (Figure 2C). Taken together, (i) either MRM analysis or immunoblot analysis comparably tracked Rac/Cdc42 and (H/K/N)Ras glucosylation in toxin-treated Caco-2 cells, (ii) the glucosylation of (H/K/N)Ras by TcdA-10463 was delayed as compared with glucosylation of Rac/Cdc42 glucosylation, and (iii) (H/K/N)Ras was not glucosylated by TcdB-10463.

**FIGURE 3:**
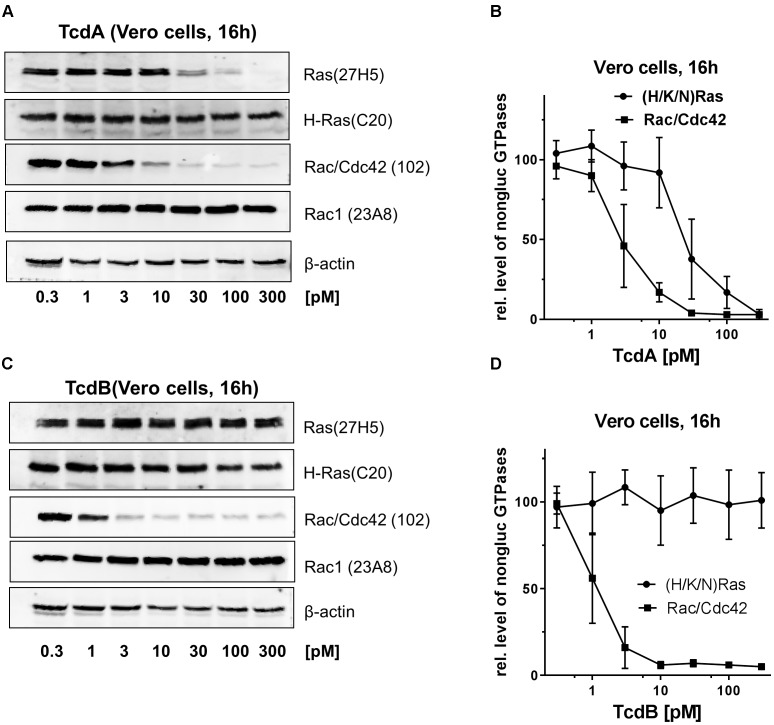
Immunoblot-based analysis of the glucosylation of (H/K/N)Ras and Rac/Cdc42 in TcdA- and TcdB-treated Vero cells. Vero cells were exposed to the indicated concentrations of native TcdA-10463 **(A,B)** and TcdB-10463 **(C,D)** for 16 h. The cellular levels of non-glucosylated (H/K/N)Ras, total H-Ras, non-glucosylated Rac/Cdc42, total Rac1, and beta-actin were analyzed by immunoblot analysis. Immunoblots were quantified using Kodak software. The amounts of non-glucosylated Rac/Cdc42 and non-glucosylated (H/K/N)Ras relative to the, respectively, levels of Rac1 and H-Ras are expressed as mean ± SD of three experiments.

The kinetics of Rac/Cdc42 and (H/K/N)Ras glucosylation were further investigated in African green monkey kidney (Vero) cells, an epithelial cell line with high sensitivity to the *C. difficile* toxins (Orth et al., 2014). In TcdA-10463-treated Vero cells, Rac/Cdc42 was about one order magnitude more efficaciously glucosylated than (H/K/N)Ras, as evaluated exploiting the Rac1(Mab 102) and the Ras(Mab 27H5) antibodies (Figures 3A,B). In contrast to Caco-2 cells, (H/K/N)Ras was completely glucosylated in TcdA-10463-treated Vero cells (Figures 3A,B). In TcdB-10463-treated Vero cells, (H/K/N)Ras was not glucosylated even at TcdB-10463 concentrations two orders of magnitude greater than those required for complete Rac/Cdc42 glucosylation (Figures 3C,D). These observation from Vero cells further excluded that (H/K/N)Ras was glucosylated by TcdB-10463.

**FIGURE 4:**
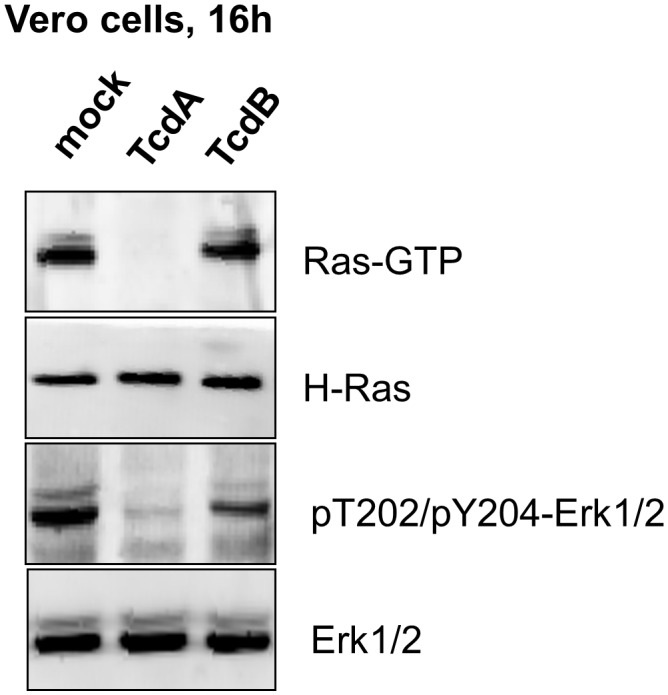
Inactivation of (H/K/N)Ras in TcdA-treated Vero cells. Vero cells were exposed to native TcdA-10463 and TcdB-10463 for 16 h. Cells were lysed and objected to effector pull-down assay exploiting the Ras-binding domain of Raf kinase. The cellular levels of GTP-loaded (H/K/N)Ras, total H-Ras, pT202/pY204-p44/42-MAP kinase (pT202/pY204-ERK1/2), and total p44/42-MAP kinase (ERK1/2) were analyzed by immunoblot analysis using the indicated antibodies.

The cellular levels of active GTP-bound Ras were next analyzed using pull-down assay exploiting the Ras binding domain of Raf kinase (van Triest and Bos, 2004). In TcdA-10463-treated Vero cells, (H/K/N)Ras was completely inactivated, as no GTP-bound active (H/K/N)Ras was detected (Figure 4). In contrast, GTP-bound active (H/K/N)Ras was present in TcdB- and mock-treated cells (Figure 4). (H/K/N)Ras activates the canonical pathways leading to activation of p44/42-MAP kinase (ERK1/2). In mock-treated Vero cells, active ERK1/2 was detected in terms of the cellular level of pT202/Y204-ERK1/2. In TcdA-10463-treated Vero cells, almost no pT202/Y204-ERK1/2 was detected (Figure 4), reflecting Ras inactivation. The level of pT202/Y204-ERK1/2 was slightly reduced in TcdB-10463-treated cells (as compared to mock-treated cells) (Figure 4). This observation reflects that – besides (H/K/N)Ras – ERK1/2 is activated by Rac1 to some extent (Niba et al., 2013). Slightly reduced levels of pERK1/2 in TcdB-treated Vero cells most likely reflected TcdB-catalyzed Rac1 glucosylation. The Ras-ERK pathway was thus completely inhibited in TcdA-treated Vero cells, reinforcing the view that (H/K/N)Ras was inactivated by TcdA.

### Enhanced Glucosylation of Substrate GTPases in the Presence of Caco-2 Lysates

**FIGURE 5:**
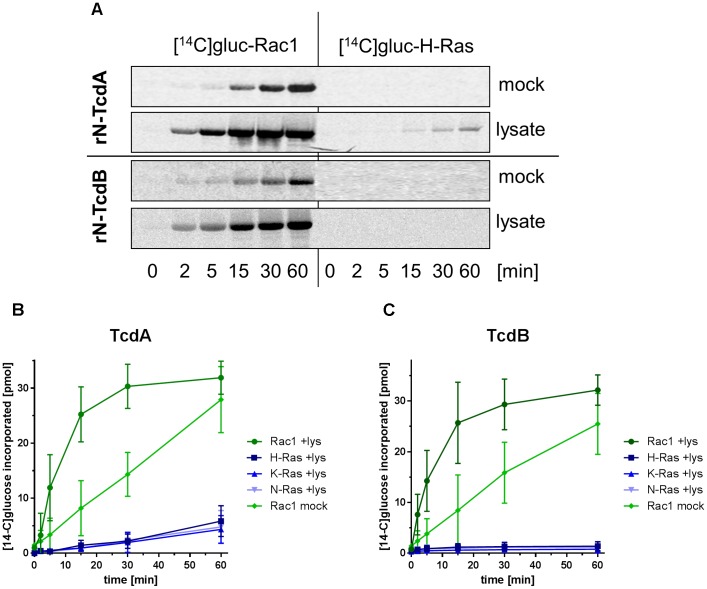
Enhanced glucosylation of substrate GTPases catalyzed by rN-TcdA and rN-TcdB in the presence of the Caco-2 lysates. GST-tagged GTPases (2 μM), UDP-[^14^C]glucose (40 μM) and recombinant rN-TcdA (3 nM, **A,B**) or rN-TcdB (0.3 nM, **A,C**) were incubated in the presence and the absence of Caco-2 lysates for the indicated times. Upon separation on SDS-PAGE, [^14^C]glucosylated GTPases were visualized by autoradiography. Signals were quantified using Kodak software and are given as mean ± SD of three experiments.

Next, GTPase glucosylation catalyzed by the recombinant glucosyltransferase domains of TcdA-10463 (rN-TcdA) and of TcdB-10463 (rN-TcdB) was re-analyzed in a cell-free system. Specifically GST-Rac1 but not GST-H-Ras was [^14^C]glucosylated by rN-TcdA and rN-TcdB applied at a relatively low toxin concentration of 0.3 nM (Figure 5A). In the presence of Caco-2 lysates (that contain the heavy and light membrane fractions), Rac1 glucosylation by rN-TcdA was strongly enhanced (Figures 5A,B). Furthermore, partial glucosylation of (H/K/N)Ras by rN-TcdA was observed in the presence of Caco-2 lysates (Figures 5A,B). Rac1 glucosylation by rN-TcdB was also enhanced in the presence of Caco-2 lysates (Figures 5A,C). However, (H/K/N)Ras glucosylation by rN-TcdB was not observed even in the presence of lysates (Figures 5A,C).

**FIGURE 6:**
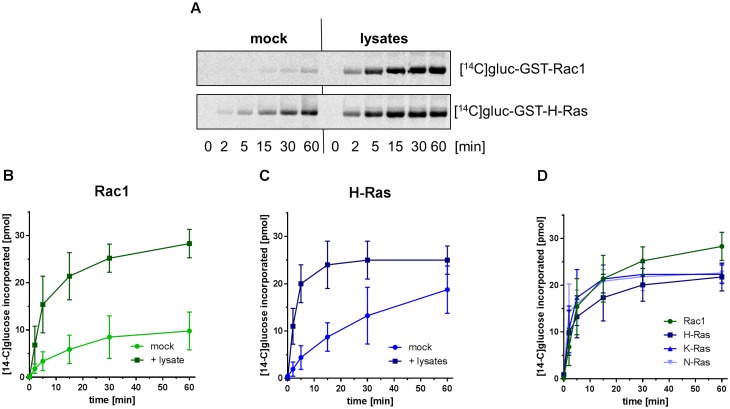
Enhanced glucosylation of substrate GTPases catalyzed by rN-TcsL in the presence of the Caco-2 lysates. GST-tagged Rac1 **(A,B)** and GST-tagged Ras-GTPases **(A,C,D)** (2 μM), UDP-[^14^C]glucose (20 μM each) and recombinant rN-TcsL (0.3 nM) were incubated in the presence and the absence of Caco-2 lysates at 37°C for the indicated times. Upon separation on SDS-PAGE, [^14^C]glucosylated GTPases were visualized by autoradiography. Signals were quantified using Kodak software and are given as mean ± SD of three experiments.

The recombinant glucosyltransferase domain of TcsL-6018 (rN-TcsL) preferably [^14^C]glucosylated GST-(H/K/N)Ras as compared with GST-Rac1 (Figure 6A), consistent with former observations (Huelsenbeck et al., 2009). The latter observation apparently contradicts above observation that (H/K/N)Ras and Rac1 were glucosylated in TcsL-treated Caco-2 cells with almost comparable kinetics (Figure 1C). To solve this apparent contradiction, the glucosylation of either Rac1 and (H/K/N)Ras by rN-TcsL was analyzed in the presence of Caco-2 lysates. The glucosylation of Rac1 (Figures 6A,B) and H-Ras (Figures 6A,C) was strongly increased in the presence of Caco-2 lysates and Rac1 and (H/K/N)Ras were glucosylated with comparable kinetics (Figures 6A,D). Comparable glucosylation of Rac1 and (H/K/N)Ras by rN-TcsL was thus not observed until Caco-2 lysates were added to the cell-free system. A cell-free system complemented with Caco-2 lysates seems to be suitable for predicting intracellular glucosylation. In sum, substrate GTPase glucosylation by either rTcdA, rTcdB and rTcsL was enhanced in a cell-free system in the presence of lysates. Under these advanced conditions, (H/K/N)Ras was preferably glucosylated by rN-TcsL, to some extent by rN-TcdA, but not by rN-TcdB.

## Discussion

Analysis of GTPase glucosylation by LCGT has widely been studied in cell-free systems to determine the specificities of LCGTs for particular small GTPases (Just et al., 1995b; Genth et al., 2014). Initial evaluation of the profiles of substrate GTPase profile of full-length TcdA-10463 and TcdB-10463 in cell-free systems has revealed that TcdA-10463 and TcdB-10463 specifically glucosylate the Rho subtype GTPases including RhoA, Rac1, and Cdc42 but not Ras subtype GTPases (Just et al., 1995a,b). It had been assumed that similar specificity pattern exist in a cell-free and a cellular context. However, mass spectrometry-based analysis revealed that TcdA glucosylates RhoA/B/C, Rac1, RhoG, and Cdc42 but also the Ras subtype GTPases Rap1/2 and (H/K/N)Ras in Caco-2 cells (Figure 1) (Zeiser et al., 2013; Junemann et al., 2017). The apparent contradiction that TcdA glucosylates Ras subtype GTPases in cellular but not in cell-free system is solved by advancing the cell-free system in two aspects: (1) Application of rN-TcdA: rN-TcdA better mimics the situation inside the cell, as the N-terminal glucosyltransferase domain of TcdA is cleaved off the delivery domain upon cell entry. rN-TcdA (applied at a relatively high concentration of 100 nM) has been shown to glucosylate a broader profile of substrate GTPases (including Ras subtype GTPases), while full-length TcdA fails to do so (Genth et al., 2014); (2) Complementation of the cell-free system with membrane-containing lysates: The substrate GTPases of the LCGTs are anchored to membranes through their C-terminally located polybasic domain and the isoprenyl residue. The 4-helix-bundle at the very N-terminus of the glucosyltransferase domain of the LCGTs mediates membrane anchoring as well (Varela Chavez et al., 2015, 2016; Craven and Lacy, 2016). Membrane anchoring of both substrate GTPases and the glucosyltransferase domain of the LCGTs facilitates enhanced glucosylation as compared with the soluble components, as substrate GTPase glucosylation catalyzed by either rN-TcdA (Figure 5B), rN-TcdB (Figure 5C), and rN-TcsL (Figure 6) was enhanced in a cell-free system upon addition of membrane-containing lysates. In particular, rN-TcdA-catalyzed glucosylation was strongly enhanced in the presence of Caco-2 lysate facilitating the detection of glucosylation of (H/K/N)Ras (Figures 5A,B).

(H/K/N)Ras is observed to be glucosylated in TcdA-treated Caco-2 cells, as evidenced by MRM analysis (Figure 1). In general, the LC-MS based MRM method exhibits a higher specificity but less sensitivity compared to the antibody detection. In terms of antibody detection, glucosylation of (H/K/N)Ras has been observed in TcdA-treated Caco-2 and Vero cells (Figures 2, 3), complementing the observations from the MRM analysis (Figures 1D,E). Ras glucosylation results in blocked Ras signaling in TcdA-treated cells, as evidenced in terms of inhibited Ras-ERK signaling (Figure 4). In contrast to TcdA, TcdB-10643-catalyzed glucosylation of Ras-GTPases was observed neither in Caco-2 cells (Figures 1, 2) nor in Vero cells (Figure 3) nor in a cell-free system (Figure 5), as analyzed in terms of MRM analysis, immunoblot analysis, and [^14^C]glucosylation. Comparable to TcdB-10463, TcdB-R20291 did also not glucosylate Ras-GTPases in Caco-2 cells (Figure 1H). In conclusion, the two TcdB isoforms tested in this study (either of which derives from *C. difficile* toxinotype A+B+ strains) do not glucosylate Ras, which leads to the prediction that TcdB-10463 and TcdB-R20291 more efficiently promote inflammatory response in cells as compared to TcdA-10463. Finally, the GTPase profile of TcdB isoforms from so called variant, toxinotype A-B+ strains (such as strain 1470) remains to be analyzed because these strains must be expected to exhibit Ras glucosylation (Huelsenbeck et al., 2007; Genth et al., 2008).

In TcsL-treated Caco-2 cells, Rac1 and (H/K/N)Ras exhibited almost comparable kinetics of glucosylation (Figure 1C). In a cell-free system, comparable glucosylation of Rac1 and (H/K/N)Ras by rN-TcsL was not observed until the cell-free system with complemented with membrane-containing lysates (Figure 6).

Upon combined treatment of Caco-2 cells with TcdA and TcdB, TcdA-catalyzed Ras glucosylation was suppressed (Figure 1F). This unexpected observation suggests that TcdB is capable of reducing TcdA uptake. TcdA enters target Caco-2 cells (but not other yet investigated cell lines) by clathrin-independent endocytosis, which requires intact actin dynamics (Robertson et al., 2009; Papatheodorou et al., 2010; Chandrasekaran et al., 2016). TcdB-induced glucosylation of Rho-GTPases results in actin depolymerization (May et al., 2013), which in turn might reduce TcdA uptake and subsequent Ras glucosyslation.

Taken together, the profiles of small GTPases glucosylated by LCGTs can be analyzed with the presented methods in cell-free systems, cultured cells, and in tissue from animal models and infected humans. The presented methods will allow the identification of new toxinotypes that exhibit different GTPase substrate profiles. Finally, observations from three independent experimental systems exclude that TcdB glucosylates Ras subtype GTPases. The observations of this study leads to the recommendation that TcdB (rather than TcdA) should be applied as tool in cell biology research to check for an involvement of Rho subtype GTPases in cellular processes.

## Materials and Methods

### Materials

Toxins: Recombinant toxins and toxin fragments as well as native toxin were used in parallel. The glucosyltransferase domains (covering amino acids 1–546) of TcsL (rN-TcsL, strain 6018) and TcdB (rN-TcdB, strain VPI10463) were expressed in *Escherichia coli* using the pGEX-2T vector system and affinity purified using Glutathion-Sepharose Beads (AP Biotech) as described (Hofmann et al., 1997). The glucosyltransferase domain (covering amino acid 1 to 1065) of TcdA (rN-TcdA, strain VPI10463) and full-length TcdA-10463, TcdB-10463, and TcdB-R20291 were expressed in the *Bacillus megaterium* expression system (MoBiTec, Germany) (Wohlan et al., 2014). Full length TcsL-6018 was produced in *C. sordellii* strain 6018 and purified yielding only one band on SDS-PAGE as previously described (Genth et al., 2000). In brief, a dialysis bag containing 900 mL of 0.9% NaCl in a total volume of 4 liters of brain heart infusion (Difco, BD Life Sciences, Heidelberg, Germany) was inoculated with 100 mL of an overnight culture of *C. sordellii*. The culture was grown under microaerophilic conditions at 37°C for 72 h. Bacteria were removed from the dialysis bag solution by centrifugation. Proteins from the culture supernatant were precipitated by ammonium sulfate at 70% saturation. The precipitated proteins were dissolved in 50 mM Tris-HCl, pH 7.5 buffer and extensively dialyzed against 50 mM Tris-HCl, pH 7.5 buffer for 24 h. The protein solution was loaded onto an anion exchange column (MonoQ, AP Biotech, New Jersey, NJ, United States). TcdA was eluted with 50 mM Tris-HCl, pH 7.5, at 150–200 mM NaCl. TcsL or TcdB were eluted at 500–600 mM NaCl. The toxins were subsequently dialyzed against buffer (50 mM Tris-HCl pH 7.5, 15 mM NaCl). Immunoblot analyses were used to identify and calculate the amount of isolated toxins.

Purification of recombinant proteins: GST-tagged Rho and Ras subtype GTPases were expressed in *E. coli* using the pGEX-2T vector system and affinity purified using Glutathion-Sepharose Beads (AP Biotech) as described (Hofmann et al., 1997).

### Cell Culture and Preparation of Lysates

African green monkey kidney (Vero) cells and human epithelial colorectal adenocarcinoma (Caco-2) cells were cultivated in Dulbecco’s modified Eagle Medium (DMEM) containing 10% fetal calf serum (FCS), 100 U/ml penicillin G and 100 μg/ml streptomycin in a humidified atmosphere containing 5% CO_2_. Cells sub-confluently seeded in 3.5-cm dishes were treated with TcdA, TcdB, and TcsL according to the indicated concentrations. Upon incubation time, the cells were rinsed with 5 ml of ice-cold phosphate-buffered saline and scraped off in 200 μl of Laemmli lysis buffer per dish. The cells were disrupted mechanically by sonification (five times on ice). The lysate were submitted to immunoblot analysis. For MRM analyses cells were washed at least three times with ice-cold phosphate buffered saline and solubilized in Tris-HCl buffer, pH 7,5 containing 20 mM NaCl and 1 mM DTT homogenized by sonification and centrifuged at 13,000 ×*g* to remove cell debris. Protein levels were determined by the method of Bradford. Crude extracts were stored at -20°C.

### Immunoblot Analysis

Cell lysates were separated on 15% polyacrylamide gels and transferred onto nitrocellulose for 2 h at 250 mA, followed by blocking with 5% (w/v) nonfat dried milk for 1 h. Blots were incubated with the appropriate primary antibody with dilution according to the manufacturers’ instructions [beta-actin(Mab AC-40, Sigma; dilution 1:5000); PAK2 (Cell signaling 2608, dilution 1:1000); phospho-S144/141-PAK1/2 (Abcam ab40795; dilution 1:2000); Rac1 (BD Transduction Laboratories 610650, clone 102; dilution 1:1000); Rac1(Millipore 05-389, clone 23A8; dilution 1:1000); Ras(Mab27H5, Cell Signaling 3339, dilution 1:200); H-Ras (C20, SantaCruz sc-520, dilution 1:200); in buffer B (50 mM Tris-HCl, pH 7.2, 150 mM NaCl, 5 mM KCl, 0.05% (w/v) Tween 20] for 18 h and subsequently for 2 h with a horseradish peroxidase-conjugated secondary antibody (mouse: Rockland 610-1034-121; dilution 1:3000; rabbit Rockland 611-1302; dilution 1:3000). For the chemiluminescence reaction, ECL Femto (Pierce) was used. The signals were analyzed densitometrically using the KODAK 1D software.

### Preparation of Caco-2 Cell Lysates

Caco-2 cells were disrupted mechanically by sonification on ice in a detergent-free lysis buffer containing 10 mM Tris/HCl (pH 7.4), 10 mM NaCl, 2 mM MgCl2, and EDTA-free protease inhibitor cocktail (Roche, Berlin, Germany). Crude cell lysates were centrifugated at 1,000 ×*g* at 4°C for 10 min to remove the nuclear fraction and intact cells. The supernatant was used as “lysate.”

### Glucosyltransferase Reaction

Recombinant Rho and Ras subtype GTPases (50 μg/mL) was incubated with either rN-TcdA, rN-TcsL or rN-TcdB in glucosylation buffer (50 mM of HEPES pH 7.4, 0.1 mM ⋅ MgCl2, 150 mM ⋅ KCl, 100 μg/mL of BSA, 20 μM of UDP-[^14^C]glucose) (Biotrend, Cologne, Germany) in a total volume of 20 μL at 37°C for the indicated times. Proteins were analyzed by 12.5% SDS-PAGE, and [14C]glucosylated Rho and Ras subtype GTPases were visualized by Phosphorimaging (Cyclone, PerkinElmer Life and Analytical Sciences, Shelton, United States).

### MRM Analysis

Multiple reaction monitoring analysis was conducted for determining glucosylation extent of small GTPases as previously described (Junemann et al., 2017). Briefly, proteins were separated by SDS-PAGE and area between 15 and 25 kDa was cut. Proteins were digested using trypsin and peptides were subjected to MRM analysis using a triple quadrupol mass spectrometer (QTRAP4000, SCIEX). Peptides were separated in a nano LC system using an Acclaim PepMap C18 column (Thermo Fisher Scientific) that was directly connected to the ion source of the mass spectrometer. For quantification acquired raw data were processed with the Skyline software (MacCoss Lab Software, Canada) (MacLean et al., 2010).

## Author Contributions

HG, JJ, AP, RG, and IJ conceived and designed the experiments. JJ, CL, IS, and A-CL performed the experiments. JJ, HG, AP, A-CL, RG, and IJ analyzed the data. JJ, AP, and HG wrote the paper.

## Conflict of Interest Statement

The authors declare that the research was conducted in the absence of any commercial or financial relationships that could be construed as a potential conflict of interest.
